# Predictors of Post-operative Mycetoma Recurrence Using Machine-Learning Algorithms: The Mycetoma Research Center Experience

**DOI:** 10.1371/journal.pntd.0005007

**Published:** 2016-10-31

**Authors:** Ali Wadal, Tusneem Ahmed Elhassan, Hajer Ahmed Zein, Manar Elsheikh Abdel-Rahman, Ahmed Hassan Fahal

**Affiliations:** 1 The Mycetoma Research Center, University of Khartoum, Khartoum, Sudan; 2 King Faisal Specialist Hospital and Research Centre, Riyadh, Saudi Arabia; 3 Faculty of Mathematical Sciences, University of Khartoum, Khartoum, Sudan; University of Tennessee, UNITED STATES

## Abstract

Post-operative recurrence in mycetoma after adequate medical and surgical treatment is common and a serious problem. It has health, socio-economic and psychological detrimental effects on patients and families. It is with this in mind, we set out to determine the predictors of post-operative recurrence in mycetoma. The study included 1013 patients with *Madurella mycetomatis* causing eumycetoma who underwent surgical excision at the Mycetoma Research Centre, Khartoum, Sudan in the period 1991–2015. The clinical records of these patients were reviewed and relevant information was collected using a pre-designed data collection sheet. The study showed, 276 patients (27.2%) of the studied population developed post-operative recurrence, 217 were males (78.6%) and 59 were females (21.4%). Their age ranged between 5 to 70 years with a mean of 32 years. The disease duration at presentation ranged between 2 months and 17 years. The majority of the patients 118 (42.8%) had mycetoma of 1 year duration. In this study, students were the most affected; 105 (38%) followed by workers 70 (25.4%), then farmers 48(17.3%). The majority of the patients were from the Central Sudan 207 (75%), Western Sudan 53 (19.2%) while 11 patients (4%) were from the Northern part. Past history of surgical intervention performed elsewhere was reported in 196 patients (71.1%). Family history of mycetoma was reported in 50 patients (18.1%). The foot was the most affected site, 245 (88.7%), followed by the hand seen in 19 (6.8%) patients and 44 (4.5%) had different sites involvement. Most of the patients 258 (93.5%) had wide local surgical excisions while 18 had major amputation. The model predicted that the certain groups have a high risk of recurrence, and these include patients with disease duration greater than 10 years and extra-pedal mycetoma. Patients with disease duration between [5–10] years, with pedal mycetoma, who had previous surgery, with positive family history and underwent wide local surgical excision. Patients with disease duration [5–10] years, with pedal mycetoma, had previous surgery, with no family history but presented with a disease size (> 10 cm), were non- farmers and underwent wide local surgical excision. Other groups are patients with disease duration (≤5 years), with pedal mycetoma, age <59 years, living in the Western /Eastern / Southern regions of the Sudan and with positive family history and had wide local surgical excision. Also included patients with disease duration (≤5 years), with pedal mycetoma, aged <59 years, living in the northern or central region, with no family history but presented with a disease size >10 cm, working as farmers or students and underwent wide local surgical excision. In conclusion, these groups of patients need special care to reduce the incidence of post-operative recurrence with its morbidity and detrimental consequences. In depth studies for the other predisposing factors for post-operative recurrence such as genetic, immunological and environmental factors are needed.

## Author Summary

Post-operative recurrence in mycetoma is a thoughtful problem. It has numerous undesirable medical, health, socio-economic and psychological impacts on the affected patients and their families, communities and health authorities in endemic regions. It is an important motive for patients to drop out follow up and treatment incompliance and hence the inclination of patients for traditional medical treatment. The factors predicating this phenomenon were not studied previously. However, patients’ characteristics and clinical presentation can partially offer an explanation. Thus the present study was set out to understand the predictive ability of some clinical factors on predicting the post-operative recurrence of eumycetoma. The present study had showed young farmers with small sized pedal mycetoma, with short disease duration, who are residing in endemic areas, with no family history and who underwent wide local excision are most likely to remain disease free. We can also concluded that, adequate surgical treatment conditions are obligatory to achieve good outcome and to reduce recurrence. Appropriate health education programmes to encourage early presentation to medical care are essential to reduce the postoperative recurrence rate with its detrimental impacts.

## Introduction

Eumycetoma is a chronic granulomatous destructive and mutilating subcutaneous fungal infection [1, 2]. The disease is endemic in many tropical and subtropical regions in what is known as the Mycetoma Belt [3, 4]. It has many medical, health, socio-economic detrimental bearings on the affected patients and communities [5, 6]. Currently there are no accurate data on its prevalence and incidence globally, likewise the infection route, susceptibility or resistance [7, 8].

The patient usually presents with small painless subcutaneous mass which gradually increases in size and spreads along the different tissue planes which eventually causes massive damage, destruction and loss of function of the affected body parts [9, 10]. The extremities are affected most but any site can be affected [11, 12].

Effective management of mycetoma depends mainly on accurate diagnosis. This in turn depends on identification of the type of mycetoma and extent of the disease through a meticulous clinical interview, clinical examinations and a battery of investigations. The later includes various imaging techniques, organism identification using grains culture, phenotypic morphological identification, molecular techniques and cyto-histopathological identification [13,14,15,16].

The management usually involves a combination of surgery and prolonged antifungal therapy [17]. The surgical treatment ranges from wide local surgical excision (WLE), repeated debridement and amputation. Early small lesions are amenable to cure with good prognosis. However, the majority of patients present late with advanced disease and such patients are difficult to cure and frequently relapse after apparently adequate treatment with a high morbidly [18, 19, 20].

Post-operative recurrence in mycetoma is a frequent problem and its explanation is an enigma. It has many impacts on the patients and health authorities in endemic areas.

With this background, this study was conducted to understand the clinical predictors of post-operative recurrence of eumycetoma. It also aims to identify the interactions between the different predictive factors in an attempt to develop a predictive model for eumycetoma post-operative recurrence based on the most important clinical factors identified. However, this study is presented with some limitation such as the retrospective and the single-center experience nature.

## Materials and Methods

This retrospective descriptive study was conducted at the Mycetoma Research Centre (MRC), Khartoum, Sudan. It included 1013 patients with confirmed *Madurella mycetomatis* eumycetoma who underwent surgical treatment in the period 1991 and 2015.

Two hundred seventy sex of these patients (27.2%) developed post-operative recurrence. The diagnosis of eumycetoma was confirmed by clinical interview, meticulous clinical examinations, ultrasound and conventional X-ray examination of the affected part, grains culture, lesion aspirates cytological examination and histopathological examination of the surgical biopsies.

The clinical records of these patients were carefully reviewed and the data was collected using a pre-designed data collection sheet.

### Statistical analysis

#### Target variable/ outcome variable

The target/outcome variable was mycetoma post-operative recurrence which is a binary variable with two levels (recurrence vs. disease-free).

### Predictive factors

The association between clinical factors and mycetoma post-operative recurrence as a target/ outcome variable was investigated. Clinical predictive factors were selected and reformatted using the available domain knowledge provided by expertise in the field of mycetoma. These factors were: age, gender, residence, disease site and duration in years, occupation, family history, previous surgery and type of surgery. These characteristics are shown in Table 1.

**Table 1:** The patients’ population characteristics.

Characteristics	Patient population No. (%)	Patients with recurrence No. (%)
Age		
<18	169 (16.7%)	41(14.8%)
[18–30]	625 (61.7%)	165(59.7%)
[31–59]	199 (19.6%)	52(18.8%)
>60	20 (02%)	18 (6.5%)
Gender		
Male	727 (71.8%)	217 (78.6%)
Female	286 (28.2%)	59 (21.4%)
Residence		
North	48 (4.7%)	11 (04%)
Central	817 (80.7%)	207 (75%)
West	117 (11.5%)	53 (19.2%)
others	31 (03%)	
Disease duration (years)		
≤5	839 (82.8%)	101(36.5%)
(5–10]	145 (14.3%)	120(43.5%)
>10	29 (03%)	55(19.9%)
Occupation		
Farmer	156 (15.4%)	48 (17.3%).
Student	398 (39%)	105 (38%)
Workers	459 (45.3%)	70 (25.4%)
Family history		
Yes	132 (13%)	50 (18.1%)
No	881(87%)	226 (81.9%)
Previous surgery		
Yes	566 (56%)	196(71.1%)
No	447(44%)	80(28.9%)
Mycetoma site		
Foot	868 (85.7%)	245 (88.7%)
Hand	84 (8.3%)	19 (6.8%)
Other	61 (06%)	12 (4.5%)
Type of surgery		
WLE	962(95%)	258 (93.5%)
Amputation	51(05%)	18 (6.5%)

### Imputation of missing data

In this study, missing data were small and arbitrary (less than 5%). Therefore, the Markov chain Monte Carlo (MCMC) method was used assuming a multivariate normality [21].

### Machine learning models

In this study, machine learning algorithms; Decision Tree (DT) and Random Forest (RF) were utilized for predicting mycetoma post-operative recurrence assuming unknown data mechanism [22].

### Data partitioning

Data was partitioned as 70% for training the algorithms with the remaining 30% of the data kept for the validation purpose. Models were trained with 5-fold cross-validation to avoid model over fitting and to ensure model stability [23].

### Model evaluation

Model performance was evaluated using model accuracy, Positive Predictive Value (PPV), Negative Predictive Value (NPV) and Area under the Receiver Characteristic Curve (AUC) [24]. AUC measures the discrimination ability of the model on predicting the class levels of target variable based on the predictive factors. It is a single scalar value that represents the excepted performance of receiver operating characteristic (ROC) curve.

## Results

### The patients’ characteristics

The study included 1013 patients with *Madurella mycetomatis* caused eumycetoma patients who underwent surgical treatment at the Mycetoma Research Centre, Khartoum, Sudan in the period 1991–2015. The study documented that, 276 patients (27.2%) developed post-operative recurrence, [Table 1].

The study population were 727 males (71.8%) and 286 females (28.2%). Their age ranged between 5 and 70 years with a median of 23 years. Most of the patients, 625 (61.7%) were in the age group 18–39 years, 199 (18.6%) in the age group 31–59 years and 169 (16.7%) were less than 18 years old at presentation. The disease duration at presentation ranged between 2 months and 17 years. The majority of patients, 839 (82.8%) had mycetoma of less than five years duration, 171 (14.3%) had mycetoma with a duration ranged between 5 to 10 years and 29 (3%) had mycetoma for more than 10 years.

In this study, students were affected most; 398 (39%) followed by farmers 156 (15.4%). The majority of the patients were from the Central Sudan 817 (80.7%), Western Sudan 117 (11.5%) while 48 patients (4.7%) were from the Northern part.

The history of previous surgical treatment performed elsewhere was reported in 566 patients (56%). Family history of mycetoma was reported in 132 patients (13%). The foot 868 (85.7%) was the most affected site followed by the hand seen in 84 patients (8.3) and 61 patients (6%) had mycetoma at different parts.

Most of the patients, 962 (95%) had wide local surgical excisions while 51 patients (5%) had major amputation. The amputation included above and below knee, below elbow and Syem’s amputations.

### The post-operative recurrence group clinical characteristic

The study showed that, 276 patients (27.2%) developed post-operative recurrence of whom 217 were males (78.6%) and 59 were females (21.4%). Their age ranged between 5 year and 70 years with a mean of 32 years [Table 1]. The disease duration at presentation ranged between 4 months and 19 years. The majority of the patients 118 (42.8%) had mycetoma of 1 year duration. In this study, students were affected most; 105 (38%), followed by workers 70 (25.4%), then farmers 48 (17.3%). The majority of the patients were from the Central Sudan 207 (75%), Western Sudan 53 (19.2%) while 11 patients (4%) were from the Northern part.

Past history of surgical intervention performed elsewhere was reported in 196 patients (71.1%). Family history of mycetoma was reported in 50 patients (18.1%). The foot was the most affected site 245 (88.7%) followed by the hand seen in 19 (6.8%) patients and 12 (4.5%) had mycetoma at different parts.

258 (93.5%) of the patients had wide local surgical excisions while the rest of patients had major amputation. Amputation included above and below knee, below elbow and Syem’s amputations.

### Machine learning algorithms model

#### Decision Tree

Decision Tree (DT) achieved an accuracy of 80%, PPV of 78%, NPV of 81% and AUC = 0.74 in training dataset. On the other hand, using the validation dataset, DT achieved an accuracy of 73%, PPV of 69%, NPV of 74% and AUC = 62%.

#### Random forest

Random forest (RF) achieved accuracy of 81%, PPV of 84%, NPV of 81% and AUC = 0.81 in training data. Using the validation dataset, the model achieved accuracy of 74%, PPV of 74%, NPV of 74% and AUC = 64%.

**Fig 1:**
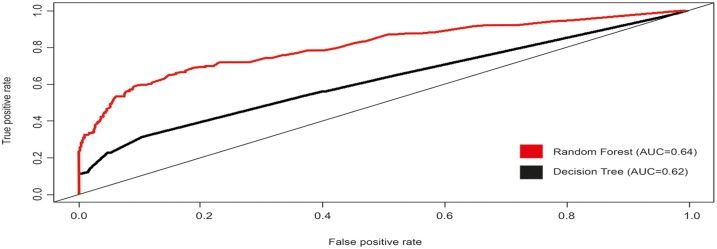
ROC curve for Decision tree and Radom forest.

Comparing the above performance measures, it could be seen that random forest outperformed the decision tree though both showed a comparable results. However, this confirms the stability of the constituent decision tree, Fig 1.

The most important output of RF is the relative importance of each predictive factor in the classification process (Gini index). Disease duration, site of mycetoma and previous surgery are the most influential factors in the recurrence classification, Table 2.

**Table 2:** Relative importance of predictors on recurrence of eumycetoma using random forest.

Predictors	Mean decrease in Gini index
Disease duration	18.37
Site	12.36
Previous surgery	9.48
Size	6.31
Residence	5.43
Age	5.37
Occupation	4.67
Family history	4.48
Medication	4.27

Comparing these results with the developed decision tree, one can find that these predictors were considered as the most important predictors because they were used in first splits in the top of DT. Therefore, the constituent decision tree and random forest showed a good performance in term of in-sample fit and out-of-sample fit.

**Fig 2:**
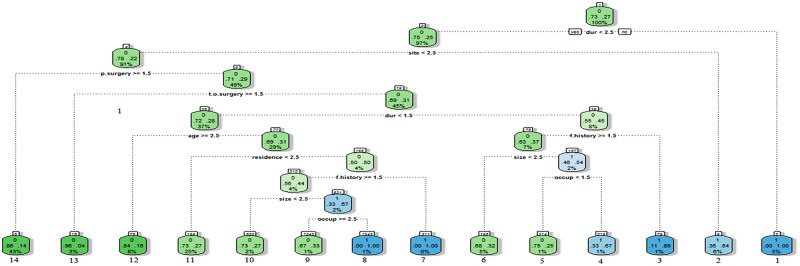
Decision classification of eumycetoma patients.

Following the designed decision tree, Fig 2, the following results were obtained

### Predicting the positive class (disease recurrence)

- Patients with disease duration greater than 10 years and extra-pedal mycetoma had high risk of recurrence, Node 1, Fig 2.
- Patients with disease duration between [5–10] years, with pedal mycetoma, had previous surgery, with positive family history and underwent wide local surgical excision had high risk of recurrence, Node 3, Fig 2.
- Patients with disease duration [5–10] years, with pedal mycetoma, had previous surgery, with no family history but presented with a disease size (> 10 cm), were non-farmers and underwent wide local surgical excision had high risk of recurrence, Node 4, Fig 2.
- Patients with disease duration (≤5 years), with pedal mycetoma, their ages <59 years, living in the Western / Eastern / Southern regions of the Sudan, with positive family history and had wide local surgical excision had high risk of recurrence, Node 7, Fig 2.
- Patients with disease duration (≤5 years), with pedal mycetoma, their ages <59 years, living in the northern or central region, with no family history but presented with a disease size >10 cm, working as farmers or students and underwent wide local surgical excision had higher risk of recurrence, Node 8, Fig 2.

### Predicting the negative class (disease free state)

- Patients with disease duration [5–10] years, with pedal mycetoma, had previous surgery, underwent local surgical excision, with no family history, with lesion size >10 cm and working as farmers were at the lowest risk to develop recurrence, Node 5, Fig 2.
- Patients with disease duration [5–10] years, with pedal mycetoma, had previous surgery, underwent local surgical excision, with no family history, with lesion size<10 were at the lowest risk to develop recurrence, Node 6, Fig 2.
- Patients with disease duration (≤5 years), pedal mycetoma, had previous surgery, underwent local surgical excision, aged <59 years, living in the western /eastern / southern regions, with no family history, with lesion size >10 and working as farmers were at the lowest risk to develop recurrence, Node 7, Fig 2.
- Patients with disease duration (≤5 years), with pedal mycetoma, had previous surgery, underwent local surgical excision, aged <59 years, living in the western /eastern / southern regions, with no family history, with lesion size<10 cm were at the lowest risk to develop recurrence, Node 8, Fig 2.
- Patients with disease duration (≤5 years), with pedal mycetoma, had previous surgery, underwent local surgical excision, aged <59 years, living in the central and northern regions were at the lowest risk to develop recurrence, Node 9, Fig 2.
- Patients with disease duration (≤5 years), with pedal mycetoma, had previous surgery, underwent local surgical excision, aged <31 years were at the lowest risk to develop recurrence, Node 10, Fig 2.
- Patients with disease duration (≤5 years), with pedal mycetoma, had previous surgery, underwent amputation were at the lowest risk to develop recurrence, Node 11, Fig 2.
- Patients with disease duration (≤5 years), pedal mycetoma, had no previous surgery were at the lowest risk to develop recurrence, Node 12, Fig 2.

## Discussion

Factors predicating post-operative recurrence were not studied previously. However, patients’ characteristics and clinical presentations can partially offer an explanation. The post-operative recurrence rate in this study was 27.2%, which is disappointing rather high. This post-operative recurrence necessitates repeated surgical excisions leading to massive tissue damage and resulting in healing by fibrosis. This is commonly associated with deformities, disabilities and loss of functions. Many efforts must be undertaken to reduce this high rate and improve the lives of these patients.

Long disease duration proved to be an important recurrence predictor in this study. Mycetoma patients have many unique clinical features and one of these is patients’ late presentation. This is due to the lack of health education, patients’ low socio-economic status and the scarcity of medical and health facilities at remote rural regions where mycetoma is endemic [1]. Thus, good health education is crucial to encourage patients early reporting for medical advice and treatment to increase cure rate and reduces recurrence rate.

The late presentation is commonly associated with massive tissue damage, deformities, destruction and fibrosis and commonly the causative organisms are usually locked in these formed fibrous tissue [25]. All these factors contributes to the incomplete and difficult surgical excisional procedures and thus high recurrence rate.

Another important finding in this study is that, patients with long standing extra-pedal mycetoma are at high risk of post-operative recurrence. Mycetoma surgery requires a bloodless field facilitated by a tourniquet for complete surgical excisions. This is not feasible in extra-pedal mycetoma and may explain the high recurrence. This is supported by the fact that patients with mycetoma in the extremities have higher chance of disease free postoperatively. Most probably the use of tourniquet and a bloodless field in extremities surgery will enable a good and adequate surgical excision. Furthermore, in long-standing, extra-pedal mycetoma, the causative organisms usually spread freely and widely in the different tissue planes thus inducing massive chronic granulomatous inflammatory tissue, fibrosis and cavitation [13]. This process will shelter and lock the organisms and hence the inability of reaching them medically or surgically.

Optimal surgical excision conditions are prerequisite for good outcome. Clinical observations highlighted that, incomplete surgical excision, performed under local anesthesia by inexperienced surgeons in a poor surgical setting in rural areas is an important cause for recurrence [1]. Local excision under local anaesthesia is contraindicated in mycetoma as wide spread disease is common and local anaesthesia will not permit complete excision. No doubt good surgical experience and reasonable equipped facilities are perquisite for good outcome.

Another high-risk group are elderly patients with pedal mycetoma of short disease duration, reside in the Western, Eastern and Southern Sudan and with positive family history of mycetoma. In this group although the disease duration was short, the age may be an important contributory factor in recurrence presumably due to decreased immunity leading to local disease spread and making complete surgical excision not feasible. Furthermore, these patients were not from mycetoma endemic areas and hence not exposed to low grade subclinical infection that boosted their immune system.

The study also showed that, patients with pedal mycetoma of 5–10 years duration with previous surgical excisions and positive family history of mycetoma were likely to develop recurrence. In this group, the previous surgery may had led to spread of the causative organisms along the different tissue planes and had induced more fibrosis and cavitation which make complete excision not feasible.

The size of the lesion at presentation seems to be a good predictor of recurrence in this study. Patients with lesions of more than 10 cm and regardless of other factors were at a high risk to develop recurrence. The wide spread of disease along different tissue planes and bone which make complete surgical excision impossible may be the explanation.

The short disease duration associated with pedal mycetoma in young age group who underwent wide local excision seems to be disease free postoperatively. This can be explained by the fact that, short disease duration is commonly associated with small lesion that can be excised completely in addition to a competent immune system in the young patients. This is logical, as with the long-standing disease the chance for the infection to spread along the different tissue planes, fibrosis, and cavitation are quite common rendering complete surgical excision impossible.

An interesting observation documented in this study is that, farmers with mycetoma lesions larger than 10 cm were more liable to be disease free postoperatively. There is no clear explanation for this but perhaps their occupation exposes them to low grade subclinical infection due to repeated exposure to the causative organisms thus enhancing their immune responses.

There is a strong association between the absence of family history of mycetoma and disease free-state observed among the studied patients. No clear explanation can be postulated but patients with family history of mycetoma may be genetically prone to develop the infection and recurrence and that make disease eradication difficult however, further studies are needed to confirm that. However, the whole family members may be sharing the same environment conditions suitable to acquire the infection and recurrence.

In conclusion, young farmers and students with pedal mycetoma of small size, with short disease duration, who were residing in endemic areas, with no family history and who underwent wide local excision were most likely to remain disease free. Adequate surgical treatment conditions are obligatory to achieve good outcome. Appropriate health education programmes to encourage early presentation to medical care are essential to reduce the postoperative recurrence rate with its detrimental impacts. In depth studies for the other causes for post-operative recurrence such as genetic, immunological and environmental factors are needed.

### Ethical statement

Ethical clearance was obtained from Soba Hospital Ethical Committee. Patients’ informed consents proved to be unnecessary in this study.

## Supporting Information

S1 Checklist (DOC)
